# Case Report: Laparoscopic Excision of a Primary Giant Splenic Hydatid Cyst: Literature Review

**DOI:** 10.4269/ajtmh.19-0400

**Published:** 2019-08-12

**Authors:** Zhu Zhuoli, Zhao Yu, Xu Liya, Liu Mingzhong, Li Shengwei

**Affiliations:** 1 Department of Hepatobiliary Surgery, Dazhou Central Hospital, Sichuan Province, China;; 2 Department of Pathology, Dazhou Central Hospital, Dazhou, Sichuan Province, China;; 3 Department of Hepatobiliary Surgery, The Second Affiliated Hospital of Chongqing Medical University, Chongqing, China

## Abstract

Cystic echinococcosis (CE), also known as hydatid cyst, is a zoonosis caused by the tapeworm *Echinococcus granulosus*. It is a common health problem in many countries. This condition predominantly affects the liver and the lungs, and the spleen to a less extent (splenic hydatid cyst, SHD). Indeed, it is estimated that SHD occurs in less than 2% of abdominal CE and 0.5–8% of CE cases. Here, we present a case of a 44-year-old Chinese woman with primary giant SHD who experienced pain in the left hypochondrium for 10 days. A combination of abdominal ultrasonography and computed tomography (CT) were used for preoperative diagnosis. Laparoscopic splenectomy was performed without any complications, and albendazole (400 mg per day) was administered postoperatively for 3 months. At 3-, 6-, 12-, and 24-month follow-up, the patient remained symptoms free, and abdominal CT found no signs of recurrence. In addition to this case, we review the previous literature on SHD treated by laparoscopy and reveal that laparoscopic approach is safe and effective for SHD. Particularly, we show that laparoscopic splenectomy is feasible for giant cysts (> 10 cm) at high risk of rupture or compressing other vital structures.

## INTRODUCTION

Cystic echinococcus (CE), also known as hydatid cyst, is a zoonosis caused by the larval form of tapeworm *Echinococcus granulosus*.^1^ The life cycle of *E. granulosus* includes a definitive host (usually dogs or related species) and an intermediate host (such as sheep, goats). Humans are also incidental intermediate hosts and are at risk for infection.^1,2^

The initial infection usually occurs in childhood with no symptoms, and the latent periods may be more than several years. This infection predominantly affects the liver followed by lungs at 70% and 25%, respectively.^1^ Splenic hydatid cyst (SHD) is a rare entity, occurring in less than 2% of abdominal CE and 0.5–8% of CE cases.^3,4^ In general, the diagnosis of SHD is based on the clinical manifestation, radiological imaging, usually combining abdominal ultrasonography (USG) and computed tomography (CT), and/or complemented by serologic tests. However, a minority of SHD cases can only be confirmed by percutaneous aspiration or exploratory laparotomy and/or histopathological examination. Surgery is the mainstay treatment for this condition, and laparoscopy may be an alternative to open surgery in some cases. Here, we report a 44-year-old female patient with a primary giant SHD excised by laparoscopic surgery and also review the previous literature on SHD treated by laparoscopy. This work is reported in line with the surgical case report (SCARE) criteria.^5^

### Case presentation.

A 44-year-old Han Chinese woman who had pain in the left hypochondrium for 10 days was admitted to the hepatobiliary surgery department at Dazhou Central Hospital, Sichuan Province, China. The patient came from a small county of Dazhou in eastern Sichuan. She moved to Tibet at the age of 32 years where she lived for 10 years. She had a history of contact with dogs and livestock at her hometown and in Tibet. On examination, the left upper abdomen appeared swollen and a mobile tender mass was palpated, but no liver was suspected. In the left upper quadrant, the spleen was enlarged 15 cm below the left costal margin and was slightly tender, without muscular defense areas or signs of peritoneal irritation. The other components of the physical examination were normal.

**Figure 1.**
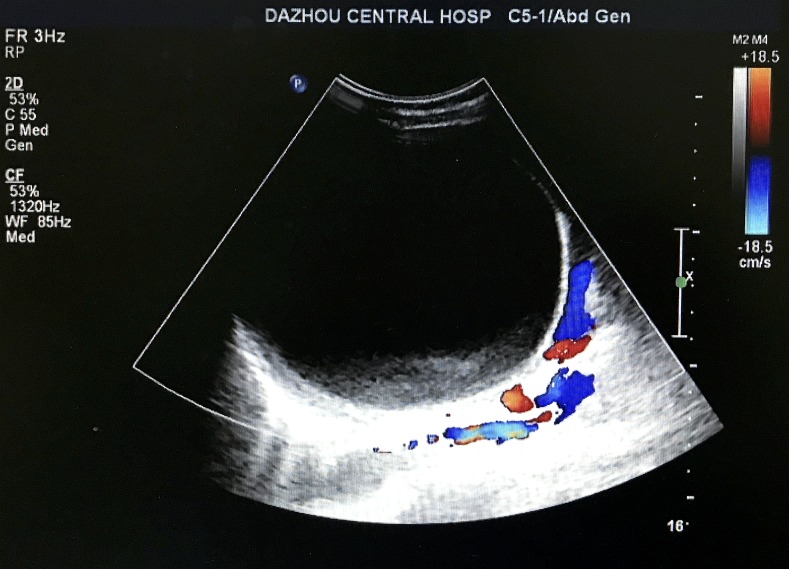
Abdominal ultrasonography showing a 13.5 × 12.1-cm–sized anechoic solitary, thin-wall cyst in the splenic parenchyma. This figure appears in color at www.ajtmh.org.

**Figure 2.**
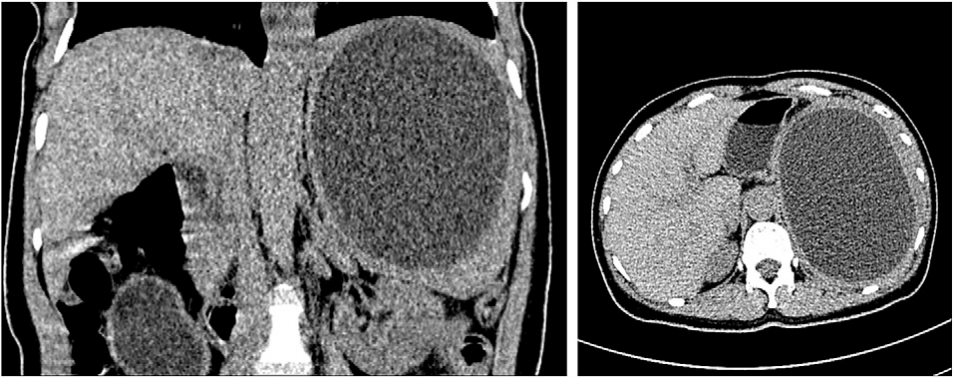
The coronal view (on left) and axial view (on right) of computed tomography showing a 15.2 × 14-cm–sized well-defined cystic lesion in the upper pole of the spleen.

Abdominal USG revealed a 13.5 × 12.1-cm–sized anechoic solitary, thin-wall cyst in the splenic parenchyma (Figure 1). Computed tomography scan showed a large well-defined hypodense cystic lesion in the upper pole of the spleen, involving almost the entire spleen and measuring 15.2 × 14 cm (Figure 2). Laboratory tests revealed mild leukocytosis (11,760 cell/mm^3^) and eosinophilia (6.8%). Other biochemical tests were in the normal range.

Based on the clinical manifestation and preoperative imaging findings, an SHD was strongly suspected. However, given the high risk of cyst rupture and obvious pressure symptoms, we did not conduct serologic tests, instead we directly performed laparoscopic splenectomy on the 2nd day of admission after communicating with the patient. A standard four-trocar placement was used to establish access ports. Pneumoperitoneum was produced in the supraumbilical area using carbon dioxide (12 mm Hg), after which a 10-mm port was placed. A 5-mm port was positioned to the left of the falciform ligament below the xiphoid and a 10-mm port was placed on the left midclavicular line as the main manipulation port. An additional 5-mm port was positioned at the inferior pole of the spleen to the left midaxillary line for injection and aspiration. An old rupture was found on the surface of the cyst in the upper pole of the spleen, which adhered to the diaphragm. Any adhesion between the cysts and neighboring organs was lysed. After locating the spleen cyst, gauzes moistened with hypertonic saline were placed around the cyst to prevent potential contamination and anaphylactic reactions. Then, a Veress needle was inserted into the cyst via the incision site, after which about 500 mL of the cyst contents were aspirated and 20% hypertonic saline was injected into the cystic cavity as a scolicidal agent. After a 5-minute waiting period, the hypertonic solution was aspirated from the cystic cavity. This cycle of injection and aspiration was repeated five times. Subsequently, the spleen was totally excised and extracted in an endobag. No bleeding points were seen, and the peritoneum was soaked with 20% hypertonic saline for 10 minutes and washed with 0.9% saline three times. Finally, a tubular drain was introduced through the left port and positioned at each of the splenic fossa and pelvic cavity.

**Figure 3.**
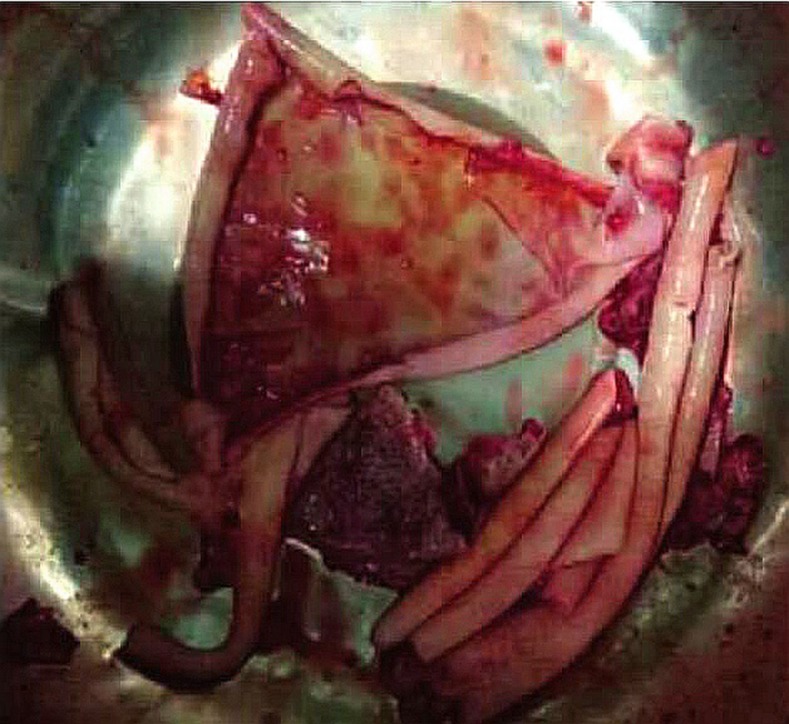
Cut section of the cyst capsule. This figure appears in color at www.ajtmh.org.

**Figure 4.**
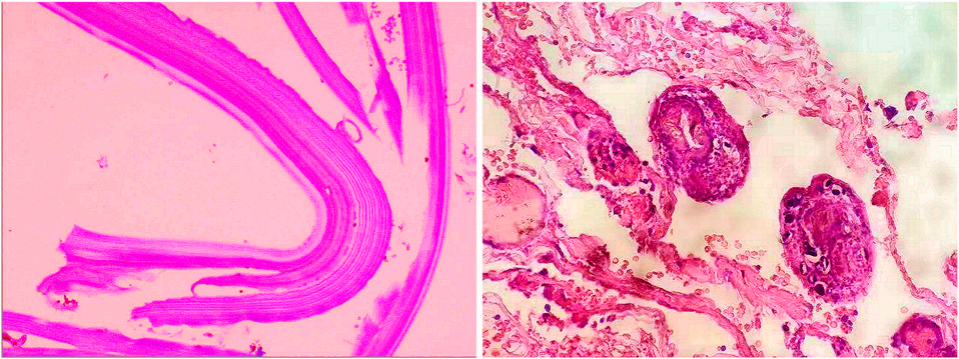
Histopathological examination showing the acellular fibrous wall (on left) and scolex (on right) of the hydatid cyst (HPE X-40). This figure appears in color at www.ajtmh.org.

**Figure 5.**
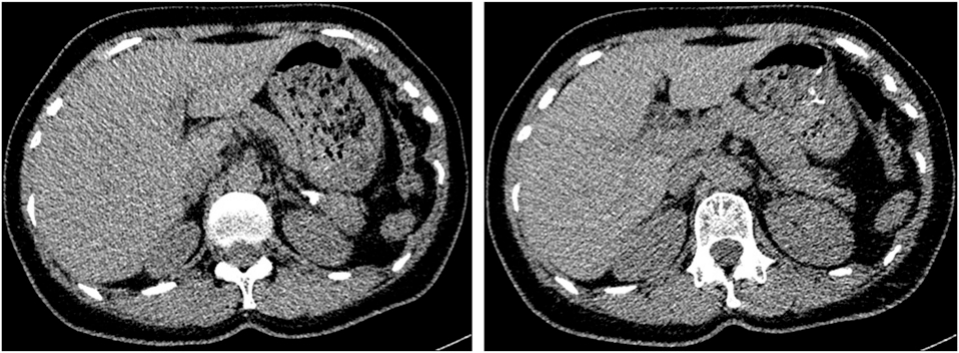
Computed tomography at postoperative 1-year follow-up showing the ligation nail image of splenic hilus underwent by endoscope linear cutting stapler (on left) and no recurrence of hydatid cyst (on right).

The cut section of the cyst capsule appeared curled and soft (Figure 3). The histopathological examination of the specimen confirmed the diagnosis of SHD (Figure 4). Postoperative recovery was uneventful and the drain was removed at day 4 after surgery, and the patient was discharged on the 6th postoperative day. Albendazole (400 mg per day) was initiated from the first postoperative day and continued for 3 months. She was followed up every 3 months. At the 12- and 24-month follow-up visits, she was asymptomatic, and her laboratory tests were normal. Both abdominal USG and CT found no disease recurrence (Figure 5).

## DISCUSSION

The WHO has included CE in the list of “neglected tropical diseases.”^6^ It is a significant public health problem in South America, the Middle East and eastern Mediterranean regions, some sub-Saharan African countries, western China, and the former Soviet Union.^7,8^ In China, Tibet and Sichuan highlands are highly endemic for CE, with rates of up to 6.8%.^9^ Cystic echinococcus affects almost any organ of the body. The liver and lung are affected in approximately 70% and 25% of patients, respectively, and other organs such as the brain, muscle, kidneys, bone, heart, and pancreas in a smaller proportion of patients.^1^ According to Gupta et al.,^10^ cysts occur in the liver (55–60%), lungs (30%), kidney (2.5%), heart (2.5%), bones (2%), muscles (1%), brain (0.5%), and spleen (1.5%). Primary involvement of spleen is even rarer and occurs in less than 2% of abdominal CE and 0.5–8% of CE cases.^3,4^

The clinical presentation of CE depends on the site and size of the cysts. Basically, the cyst grows faster in the lungs, with reaching larger sizes, and slower in the liver, spleen, and kidneys.^11^ Approximately 30% of patients with SHD are asymptomatic, and the detection of this disease is incidental in most cases. When the size of the cyst is sufficiently large, patients may present with upper left abdominal pain and/or a palpable abdominal mass.^12,13^ For CE including SHD, a combination of imaging and serological tests may be effective in diagnosing the disease, especially for patients living in endemic areas.^14^ Hydatid cysts may be visualized and evaluated with USG, CT, or magnetic resonance imaging (MRI). Ultrasonography is the first-line option for the screening of abdominal hydatidosis,^15^ its sensitivity for evaluating CE is 90–95% and specificity is 93–100%.^16–18^ Many reports suggest that CT has higher overall sensitivity than USG (95–100%).^16,19,20^ Computed tomography is the most effective tool for determining the number, size, and anatomic location of cysts, is better than USG in detecting extra-hepatic cysts, and may also be used to monitor the lesions during therapy and detecting recurrences.^21,22^ It is also superior to USG in detecting complications such as infection.^23^ It has been found that MRI has some advantages in terms of detecting obscure cysts missed by USG and CT and of providing cystic pathological features. However, it is usually avoided because of its high costs.^22,24^

Serology is useful for primary diagnosis and for evaluating response to treatment. Common serologic tests include ELISA, indirect hemagglutination (IHA), immunoelectrophoresis, latex agglutination test, and Western blot.^18,22^ ELISA appears to be the most sensitive and specific among the available assays.^25^ Force et al.^26^ compared eight serologic tests among 131 patients with *E. granulosus* infection. They found that IgG ELISA was the most sensitive (94%) and specific (99%) for the majority of cyst locations. Akbulut et al.,^27^ who reviewed the 10 previous studies, also demonstrated that ELISA was the best method for diagnosing SHD, with a sensitivity of 71.4–100%. Wuestenberg et al.^18^ reported that IHA had a sensitivity of 50–100% and specificity of 83–88%. Eris et al.^22^ pointed that the rate of IHA positivity was 57% in primary SHDs and 100% in secondary cases. Therefore, combining ELISA and IHA may increase the sensitivity. These tests, however, may be negative and limited because of the capsule isolation of parasite from the host’s immune system.^28^

Management options for CE include surgery, percutaneous management, drug therapy, and observation stage.^29,30^ According to the WHO diagnostic classification, regarding CE with SHD, stage CE-1 and CE-3a cysts have a single compartment. Such cysts that are < 5 cm may be treated with albendazole alone. Cysts that are > 5 cm may be treated with puncture, aspiration, injection, and reaspiration (PAIR) alone or in combination with albendazole. Management for stage CE-2 and CE-3b cysts, which have many compartments, requires either modified catheterization technique or surgery (with adjunctive drug therapy). Stages CE-4 and CE-5 are inactive cysts that can be managed with observation.^29,31^ The optimal choice between these approaches is uncertain because of the low level of evidence. As a traditional therapeutic modality, surgery may be effective in some situations for example, single cyst diameter > 10 cm, superficial cyst at risk of rupture, rupture cyst, cysts compressing vital structures, cysts with secondary infection or hemorrhage, and cysts with many daughter vesicles.^29,32^ The radical surgical options used for the management of SHD > 5 cm are mainly divided into splenectomy and spleen-saving procedures (e.g., partial splenectomy, enucleation, deroofing with omentoplasty, internal drainage with cystojejunostomy, or external drainage).^33^ Since the first case of SHD treated by laparoscopy was reported in 1997,^34^ several reports have demonstrated the safety of laparoscopy for SHD treatment compared with an open approach.^35^ However, sepsis-related mortality rates associated with total splenectomy are 4% and 1.9% in children and adults, respectively.^36,37^ Thus, surgical approaches that preserve the spleen are preferred. Some studies have revealed that no obvious differences were observed in disease recurrence, postoperative hospitalization, or complications when total splenectomy or spleen-saving procedures were implemented.^27,38^ Arikanoglu et al. and Eris et al. stated that spleen-saving surgery for SHD was more appropriate in young patients and those with peripherally located small cysts, and splenectomy was particularly effective in cysts showing multiple, larger, centrally located, and other organ involvement.^22,33^ The goal of surgery is to clear the cyst and eliminate residual cavity. Dervenis et al.^39^ pointed that laparoscopy may increase the risk of spillage due to elevated intra-abdominal pressure caused by pneumoperitoneum. To avoid fluid spillage, a protoscolicidal agent such as hypertonic saline, cetrimide–chlorhexidine, or polidocanol should be injected into the cyst, the surgical field should be protected with pads soaked in protoscolicidal agents and the peritoneum should be washed with hypertonic saline if spillage occurs. It has been reported that perioperative adjunctive therapy with albendazole (or mebendazole) can soften the cyst, facilitating removal and reducing the risk of disease recurrence by inactivating protoscolices.^40–42^ The WHO suggests that albendazole (or mebendazole) therapy should be initiated 4–30 days preoperatively and continued for at least 1 month (albendazole) or 3 months (mebendazole) postoperatively.^43,44^ However, the optimal treatment duration is unclear because of lack of sufficient evidence.

**Figure 6.**
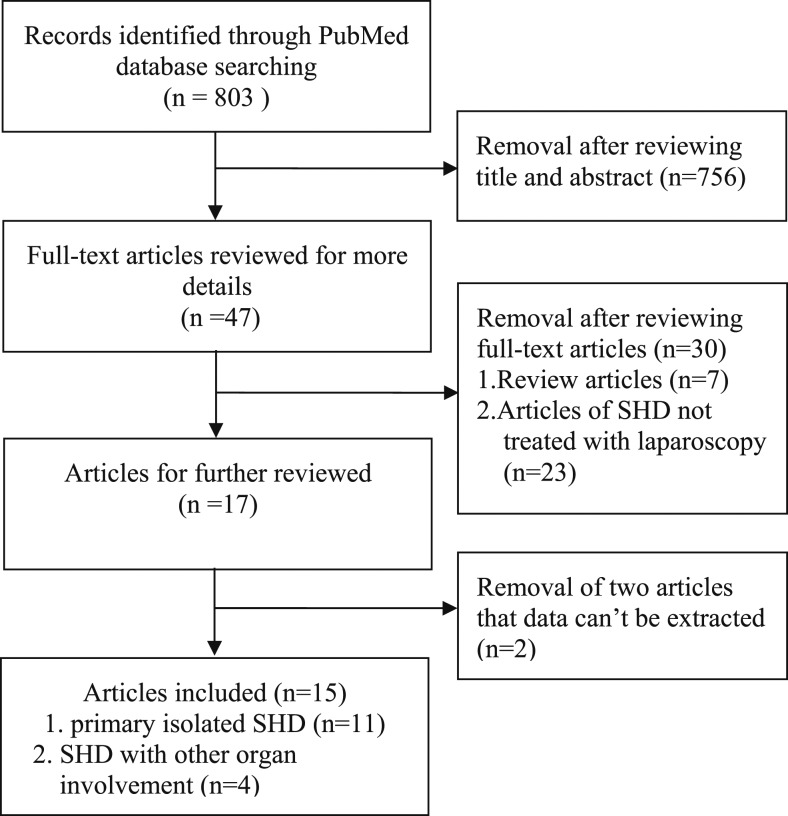
Flow diagram of studies selection process. SHD, splenic hydatid cyst.

We searched the PubMed database from the inception through February 20, 2019, using the following keywords “splenic hydatid cyst”, “hydatidosis”, “echinococcosis”, “spleen”, and their synonyms to identify studies in which SHD was treated by laparoscopy and summarize the characteristics of the clinical data. The search strategy initially generated 803 relevant articles. After reading the titles and abstracts, 756 articles were excluded. A total of 47 full-text articles were reviewed in detail. Finally, 15 articles^33–35,45–56^ were included in the analysis, of which 11 articles reported primary isolated SHD and four articles reported spleen and other organs involvement. Figure 6 shows the selection process for the studies. The clinical data of the 15 SHD patients included in the analysis is shown in Table 1. Among the patients (female/male, 8, 53.3%/7, 46.7%; children < 18 years/ adults, 4, 26.7%/11, 73.3%; age range, 6–75 years; mean age, 38.27 years), 11 (73.3%) presented with abdominal pain, among whom seven had pain in the left upper abdomen. All patients underwent preoperative diagnosis by abdominal USG, CT alone, or the combination, and among whom eight were supplemented by serologic tests, with four positive and four negative results. The imaging findings revealed an anechoic (hypodense) unilocular cystic lesion with a calcified wall, with or without daughter vesicles in the organs. Total splenectomy by laparoscopy was performed in six patients (one child and five adults; cysts location, three patients in central segment of spleen, one near the hilar region in the upper pole of spleen, one in the upper pole with the left ovary involvement, and one not mentioned; cysts sizes range: 3.2–16.5 cm) and spleen-saving surgery was performed in nine patients (three children and six adults; cyst location; in the upper pole of the spleen in three patients, in the lower pole in two patients, in the peripheral of the spleen in one patient, and in the upper pole with liver involvement in three patients; cyst sizes range: 3.5–18 cm). There were no significant differences in cysts sizes between the two surgical approaches. All patients, except one, suffered from hemorrhage and intra-abdominal abscess, but no complications were reported. Eleven cases reported perioperative treatment with albendazole, with huge variation in the duration of use. No recurrences were observed in all cases during the 3 months to 8 years follow-up, of which only one study reported a positive IHA test.

**Table 1:** Clinical data of patients with splenic hydatid cyst treated by laparoscopic surgery

	Author	Year	Country	Gender	Age	Symptoms	Localization	Cyst’s size (cm)	Cyst type	Diagnostic studies
Primary isolated splenic hydatid cyst	Arce et al.^35^	2016	Puru	F	75	Left hypochondrium pain	Upper polar segment	16.5 × 13	Cyst with daughter vesicles and calcified wall	CT + positive serology (EITB)
	Purushottam et al.^45^	2016	India	F	61	Left hypochondrium pain with dyspepsia and heart burn	Medial aspect of splenic parenchyma	4.3 × 3.2	Cyst with calcified wall and internal floating membranes	USG + CT + negative serology (IgG)
	Busić et al.^46^	2015	Croatia	F	75	NS	Upper polar segment	18 × 16 × 12	Cyst with daughter vesicles	USG + CT + positive serology (ELISA + IgG)
	Arikanoglu et al.^33^	2012	Turkey	F	32	Left upper abdominal pain and fever	Peripheral of the spleen	6 × 5	NS	USG + CT
	Vezakis et al.^47^	2012	Greece	M	44	Abdominal pain	NS	NS	Cyst with calcified wall	USG + CT
	Singal et al.^48^	2011	India	M	14	Left hypochondrium pain	Hilar region in the upper polar segment	9 × 7	Cyst with daughter vesicles	USG
	Akkoyun et al.^49^	2011	Turkey	F	10	Abdominal pain	Lower polar segment	7 × 6 × 6	Cyst with daughter vesicles	USG
	Polat et al.^50^	2009	Turkey	F	38	Left upper quadrant pain and abdominal discomfort	Upper polar segment	6 × 7	Cyst with daughter vesicles	USG + CT + positive Casoni test
	Diaconescu et al.^51^	2008	Romania	M	44	Asymptomatic mass in the left hypochondrium	Central segment	12.5 × 12.5	Unilocular cyst	USG + CT
	Gharaibeh ^52^	2001	Jordan	M	45	Left upper quadrant heaviness	Lower polar segment	8 × 12	Unilocular cyst	CT + negative serology (IHA)
	Ballaux et al.^34^	1997	Belgium	M	26	Diffuse urticarial	Central segment	16.5 × 13	Partially calcified unruptured cyst with daughter vesicles	CT + positive serology (RAST)
Non-isolated splenic hydatid cyst	Doğan et al.^53^	2013	Turkey	F	19	Abdominal pain	The left ovary; upper polar of the spleen	Ovary 4.4 × 4.3; spleen 6.5 × 6.5	Cyst with daughter vesicles	USG + CT
	Tomuş et al.^54^	2013	Romania	F	16	Upper abdominal pain	Liver segment 8; upper polar of the spleen	Liver 7 × 6.5; spleen 8.5 × 8.5	Unilocular cyst	CT + positive serology (IgG)
	Mishra et al.^55^	2010	India	M	6	Abdominal pain and fever	Liver segment 5 and 6; upper polar of the spleen	Liver 7.5 × 7.0 × 4.5; spleen 5.4 × 5 × 3.5	Cyst with daughter vesicles	USG + CT + negative serology (ELISA)
	Georgescu et al.^56^	2004	Romania	M	69	Irritative dry cough	Lung, liver and spleen	Spleen 11 × 11	NS	USG + thoracolaparoscopy

	Author	Surgery method	Complications	Hospital stay	Presurgery treatment time	Postsurgery treatment time	Follow-up recurrence
Primary isolated splenic hydatid cyst	Arce et al.^35^	Partial cystectomy	No	10 days after surgery	Albendazole 400 mg twice/day for 5 days	Albendazole 400 mg twice/day for 3 months	3 months
							No
	Purushottam et al.^45^	Splenectomy	No	NS	NS	NS	6 months
							No
	Busić et al.^46^	Partial cystectomy	No	5 days after surgery	Albendazole for 1 month	NS	6 months
							No
	Arikanoglu et al.^33^	Partial cystectomy	Hemorrhage AND intra-abdominal abscess	15 days	NS	NS	NS
	Vezakis et al.^47^	Splenectomy	No	3 days after surgery	NS	Albendazole 10 mg/kg/day for 3 months	12 months
							No
	Singal et al.^48^	Splenectomy	NS	NS	Albendazole (NS)	NS	NS
	Akkoyun et al.^49^	Partial cystectomy	No	2 days after surgery	Albendazole 10 mg/kg/day (NS)	Albendazole 10 mg/kg/day (NS)	21 months
							No
	Polat et al.^50^	Partial cystectomy	No	2 days after surgery	Albendazole 400 mg/day for 28 days	NS	8 years
							No
	Diaconescu et al.^51^	Splenectomy	No	NS	NS	Albendazole 20 mL/day for 3 months	24 months
							No
	Gharaibeh ^52^	Partial cystectomy	No	3 days after surgery	Albendazole 400 mg twice/day (NS)	Albendazole 400 mg twice/day for 2 months	13 months
							No
	Ballaux et al.^34^	Splenectomy	No	4 days after surgery	NS	NS	NS
Non-isolated splenic hydatid cyst	Doğan et al.^53^	Cystectomy, splenectomy	No	6 days after surgery	No	Albendazole 15 mg/kg/day for 3 months	3 months
							Positive IHA test
	Tomuş et al.^54^	Partial cystectomy	No	4 days after surgery	Albendazole 10 mg/kg/day for 5 days	Albendazole 10 mg/kg/day for 3 months	12 months
							No
	Mishra et al.^55^	Partial cystectomy	No	3 days after surgery	Albendazole 10 mg/kg/day (NS)	Albendazole 10 mg/kg/day for 3 months	21 months
							No
	Georgescu et al.^56^	Partial cystectomy	No	14 days after surgery	Albendazole 10 mg/kg/day for 10 days	Albendazole 10 mg/kg/day for 3 months	NS

CT = computed tomography; EITB = enzyme-linked immunoelectrotransfer blot assay; F = female; IHA = indirect hemagglutination; M = male; NS = nonspecific; USG = ultrasonography; RAST = echinococcal radioallergosorbent test.

In our case, according to the medical history and imaging findings, which were even though different from the typical CE features described in most previous studies and WHO classification of CE-1 (“snow flake sign” in USG),^31^ the diagnosis of SHD has been established. Thus, we did not conduct serologic tests as they were not considered to be necessary. In terms of management options, because the cyst was giant, superficially located in the upper pole of spleen, and accompanied with high risk of rupture that may cause severe complications (i.e., anaphylactic reactions, shock, or even death),^57^ laparoscopic splenectomy was initially performed, which was further supported by intraoperative examination of the old ruptured cyst. Ultimately, pathological examination confirmed the diagnosis of SHD. After surgery, albendazole therapy (400 mg per day) was administered for 3 months in accordance with the WHO recommendations, and the patient had no specific complaints. Postoperative follow-up were regularly performed at 3, 6, 12, and 24 months. The patient was symptoms free and had no signs of disease recurrence. Overall, based on previous literature, WHO guidelines, and this case, we suggest a spleen-saving surgery by laparoscopy or other minimally invasive technologies (e.g., PAIR) for SHD < 5 cm, and ≥ 5 cm with a single compartment or daughter vesicles, or located in the spleen periphery in younger cases. In addition, laparoscopic splenectomy is suitable for SHD > 5 cm, particularly > 10 cm, with superficial cyst at risk of rupture or located in the central segment of the spleen, or cyst compressing vital structures or with secondary infection or hemorrhage and multiple cysts due to inoculation or via secondary spread from other organs.

## CONCLUSION

Although SHD is rare, its diagnosis can usually be established based on the history of the patient such as living in an endemic area, and symptoms of CE examined by abdominal USG and/or CT. For a certain type of CE (i.e., CE-1), it can be diagnosed by an additional serologic test, abdominal exploration, percutaneous aspiration, or biopsy. The present case study and the literature review indicate that laparoscopy for SHD is safe and effective and seems to be a good alternative to open procedure. Moreover, total splenectomy is suitable for giant cysts (> 10 cm) at high risk of rupture or compressing other vital structures.
